# Impact of a physical activity program on the health-related quality of life in pediatric cancer patients: a study protocol

**DOI:** 10.3389/fspor.2025.1559431

**Published:** 2025-06-25

**Authors:** Gustavo Galárraga, Rebecca Wurtz, Marcos Di Stefano, Libet Bosch, Erika Villanueva, Karen Unda, Yunqi Yu, Carmen Apolo, Camila Coloma, José Francisco López-Gil

**Affiliations:** ^1^ School of Public Health, University of Minnesota, Minneapolis, MN, United States; ^2^ Hospital Sociedad de Lucha Contra el Cáncer (SOLCA), Quito, Ecuador; ^3^ School of Medicine, Universidad San Francisco de Quito, Quito, Ecuador; ^4^ Oncohematology Service, Hospital Pediátrico Baca Ortiz, Quito, Ecuador; ^5^ School of Medicine, Universidad Espíritu Santo, Samborondón, Ecuador; ^6^ Vicerrectoría de Investigación y Postgrado, Universidad de Los Lagos, Osorno, Chile

**Keywords:** physical activity, quality of life, pediatric cancer, physical activity programs, lifestyle

## Abstract

Physical activity is essential for the health and well-being of children and adolescents. However, those living with cancer often experience reduced physical activity levels, which negatively impact their functional capacity and health-related quality of life (HRQoL). In Ecuador, data from the National Institute of Statistics and Census (2021) reveal that 20% of children engage in less than one hour of physical activity per week, highlighting the need for targeted interventions. While previous studies demonstrate that supervised exercise programs can improve physical and psychosocial health outcomes in pediatric oncology patients, no evidence exists from Ecuador to assess the effects of such programs. This study will examine the impact of a structured physical activity program on the HRQoL of pediatric cancer patients in Quito, Ecuador. Using a case-control design, 90 participants will be randomized into two groups: an intervention group receiving a 10-week, supervised physical activity program and a control group with no exercise intervention and standard care, with additional follow-up if deemed necessary by the social worker. HRQoL surveys using a standardized instrument will be administered to all participants (those in the intervention group and those in the control group) at baseline, post intervention, and follow-up to assess changes over time. This research will address a critical public health gap by exploring how physical activity can mitigate the adverse effects of cancer treatment, improve physical and emotional health, and enhance the HRQoL in pediatric oncology patients. Findings will contribute valuable insights for integrating physical activity into pediatric oncology care in low- and middle-income countries.

## Introduction

Physical activity plays a critical role in the growth and overall well-being of children and adolescents (1). However, those living with cancer face significant barriers to maintaining adequate physical activity levels (1). Regular physical activity has been shown to yield numerous benefits, including improvements in health-related fitness, fatigue management, neurocognitive functioning, and psychosocial well-being (2). Despite these advantages, children undergoing cancer treatment often experience reduced levels of physical activity compared to their healthy peers, which may negatively impact their health-related quality of life (HRQoL) (19, 20).

In Ecuador, there is a notable gap in research examining the impact of physical activity on pediatric cancer patients. National statistics underscore the importance of addressing this issue, as in 2021 National Institute of Statistics and Census of Ecuador reported that 3.4 million children and adolescents aged 5–17 did not meet recommended physical activity levels (3). Additionally, 20% of children engage in less than an hour of physical activity per week (4). This lack of physical activity is even more pronounced among children with cancer. Studies have shown that children undergoing chemotherapy are not only less active than their healthy peers but also exhibit lower functional capacity and diminished HRQoL (19, 20).

Globally, pediatric cancer affects approximately 400,000 children and adolescents aged 0–19 annually, with nearly 30,000 cases occurring in Latin America and the Caribbean (5, 6). In Ecuador, an estimated 1,027 new cases of pediatric cancer are diagnosed each year, with 401 deaths reported in individuals under 20 years old (6). The most common cancers in this population include leukemia, lymphoma, and central nervous system tumors (6). While advancements in cancer treatment have improved survival rates in high-income countries, less than 30% of children in low- and middle-income countries achieve remission due to limited access to comprehensive care (6).

Cancer treatments such as chemotherapy, surgery, and radiotherapy can lead to adverse effects, including reduced muscle strength, lower bone density, and fatigue (7). Historically, children undergoing treatment were advised to rest extensively; however, evidence now suggests that physical inactivity exacerbates these conditions (7). For children with cancer, structured physical activity programs have been shown to help mitigate these adverse effects and restore compromised aspects of physical health and well-being (1, 7).

Both the World Health Organization (WHO) and the U.S. Department of Health and Human Services recommend that children and adolescents engage in at least 60 min of moderate to vigorous physical activity daily (8, 21). These guidelines emphasize aerobic, muscle-strengthening, and bone-strengthening activities tailored to the child's age and enjoyment levels (8). Nevertheless, studies indicate that children with cancer often struggle to meet these guidelines, further highlighting the importance of interventions aimed at promoting physical activity (9).

Research on physical activity interventions for pediatric oncology patients demonstrates significant benefits. For instance, a 16-week program led to improvements in physical health, emotional well-being, and overall HRQoL (10, 11). By the eighth week, participants also showed enhanced social functioning and reduced fatigue (10). Similarly, studies have reported improvements in psychosocial functioning, with marked differences in emotional health scores (11). These findings underscore the potential of physical activity as a complementary therapy to reduce the adverse effects of oncological treatments and enhance HRQoL during and after treatment (12).

Despite these promising outcomes, pediatric cancer patients in Ecuador face unique challenges that limit their participation in physical activity. This study will aim to address these challenges by assessing the impact of a structured physical activity program on the HRQoL of pediatric cancer patients in Quito, Ecuador. By focusing on a population often overlooked in public health research, this study seeks to provide evidence-based insights into the role of physical activity in supporting the holistic well-being of children living with cancer.

## Material and methods

### Design

This study is designed as a randomized controlled trial to investigate the impact of a structured exercise program on the HRQoL of pediatric cancer patients. A total of 90 participants will be recruited and randomly assigned to either the intervention group or the control group.

The intervention group will participate in a 10-week structured physical activity program consisting of 120 min of supervised exercise per week, led by a team of physiotherapists and physical activity professionals from Hospital Sociedad de Lucha Contra el Cáncer del Ecuador (SOLCA) Quito. Meanwhile, the control group will not participate in any organized physical activity program but will continue with their usual activities. As part of the evaluation process, two versions of the Pediatric Quality of Life Inventory (PedsQL) survey will be administered: one tailored for children aged 8–12 and another for adolescents aged 13–18. Both groups will complete their respective surveys at three time points—baseline (week 0), post intervention (week 11), and follow-up (week 24). This approach allows for the assessment of changes in HRQoL over time and enables comparative analysis between the two age groups.

### Ethics

The study protocol has been reviewed and approved by the Cancer Committee and the Institutional Review Board (IRB) of the University of Minnesota and the Ethics Committee of the Universidad San Francisco de Quito (Quito, Ecuador) (ID: 2024-068M). The research will strictly adhere to the ethical principles outlined in the Declaration of Helsinki and comply with national and international guidelines for studies involving human participants. Written informed consent will be obtained from all the legal guardians, and assent will be sought from the children, as appropriate for their age and cognitive ability. The study is registered on ClinicalTrials.gov identifier NCT06813950.

### Study team

The study team will consist of a interdisciplinary group of professionals, including:

• A medical oncologist, responsible for overseeing the clinical care of participants and ensuring the safety of interventions.
• A social worker, providing psychosocial support to participants and their families.
• A physiotherapist, designing and supervising physical activity sessions.
• A principal investigator, coordinating the study and ensuring compliance with the protocol.
• Physical education professionals, implementing the exercise program and monitoring participant engagement.

This diverse team will ensure a holistic approach to the intervention, combining expertise in clinical care, physical activity, and participant well-being (Supplementary Table S1).

### Main outcome

This research focuses on assessing the HRQoL of pediatric cancer patients, emphasizing the relationship between structured exercise programs and improvements in well-being. HRQoL is defined, as per Dac and Abhishek, as the overall well-being of individuals, incorporating both positive and negative aspects of their lives at a specific point in time (13). Key dimensions include physical, mental, and spiritual health; relationships; education; sense of security; and general well-being (13).

To evaluate HRQoL, the study will use the PedsQL, specifically the PedsQL 3.0 Cancer Module, a validated instrument for pediatric cancer patients aged 2 to 18 years. Two age-appropriate versions will be administered, one for children aged 8–12 and another for adolescents aged 13- 18. This module comprises 20 questions addressing physical, emotional, and social functioning, as well as cancer treatment-related symptoms. Participants will self-report their responses using a four-point Likert scale, with options ranging from “never” (0) to “almost always” (4). For example: “In the past month, how often have you felt tired?”; “In the past month, how often have you experienced pain or discomfort?”.

### Collection of demographic data

Demographic data will be collected during the enrollment phase by medical oncologists from both Hospital SOLCA Quito and Hospital Baca Ortiz. The variables collected will include:

• Age: Retrieved from medical records or, if unavailable, verified using the participant's identification document. The participant's age will be recorded based on the enrollment date documented in the consent and assent forms.
• Sex: Recorded as indicated in the medical records.

### Collection of clinical data

Clinical data will be collected during the enrollment phase by medical oncologists at both recruitment sites. The variables include cancer type and treatment status. Cancer type will be classified according to the International Classification of Diseases, 10th Revision (ICD-10). Treatment status will be determined through a review of medical records, with participants categorized as either “regular follow-up”, referring to patients in post-treatment monitoring, or “undergoing treatment”, referring to patients actively receiving oncological therapy. This information will be used to apply the inclusion and exclusion criteria.

The body weight of the participants will be measured via an electronic scale (with an accuracy of 0.1 kg), whereas height will be determined via a portable height rod with an accuracy of 0.1 cm. The calculation of body mass index involves dividing an individual's body weight (measured in kilograms) by the square of their height (measured in meters). Additionally, the body mass index *z* score is determined via the age-specific and sex-specific thresholds established by the WHO (21).

### Collection of lifestyle data

Physical activity and sedentary habits will be determined using the Activity Profile – Spain – Version for Latin America (YAP-SL), a self-report tool consisting of 15 items, will be used to gather data on physical activity levels and sedentary behaviors for a 7-day recall period (23). The Spanish -language version validated for Latin America YAP questionnaire will be used (23). This instrument employs a 5-point Likert scale and is organized into three distinct sections: (1) school-related activities, (2) out-of-school activities, and (3) sedentary behaviors. First, school-related activities encompass transportation to and from the educational institution, participation in physical education classes, and engagement during lunch and recess periods. Second, out-of-school activities include those occurring prior to the school day, immediately following school hours, during the evening, and throughout the weekends, specifically on Saturday and Sunday. Third, sedentary habits include the duration of activities such as watching television, playing video games, using computers, and engaging with cell phones, in addition to a general measure of sedentary time. Additionally, recreational screen time will be assessed by asking adolescents to declare the time that they spent in different sedentary screen-based pursuits. The following questions will be asked for weekdays and weekends: (a) “How many hours a day, in your free time, do you usually spend watching TV, videos (including YouTube or similar services), DVDs, and other entertainment on a screen?”, (b) “How many hours a day, in your free time, do you usually spend playing games on a computer, games console, tablet, smartphone or other electronic device (not including moving or fitness games)?”, and (c) “How many hours a day, in your free time, do you usually spend using electronic devices such as computers, tablets or smartphones for other purposes (e.g., homework, emailing, tweeting, Facebook, chatting, browsing the internet)?”. A weighted sum of the three questions will be calculated (i.e., 5 weekdays and 2 weekend days).

Sleep duration will be assessed by asking about bedtime and wake-up time on both weekdays and weekends. For older children (8–18 years old), these data are self-reported with the following questions: “At what time do you usually go to bed?” and “At what time do you usually wake up?”. The mean daily sleep duration for each participant will subsequently be calculated on the basis of these responses via the following formula: [(average nocturnal sleep duration on weekdays × 5) + (average nocturnal sleep duration on weekends × 2)]/7. The responses were subsequently classified as “meeting sleep guidelines” if the participants presented the following sleep duration ranges according to age: 8–10 h for adolescents, 9–11 h for school-age children, 10–13 h for preschoolers, and 11–14 h for toddlers.

### Statistical analysis

The data from the PedsQL surveys will first undergo descriptive analysis by age group surveys for children and adolescents. Numerical variables will be summarized as mean and standard deviation for variables with a normal distribution or median and interquartile range for non-normally distributed variables. Categorical variables will be summarized as absolute and relative frequencies. This descriptive approach will provide an overview of the data and support subsequent analyses (see Supplementary Table 2 for details on variables and survey items).

To evaluate the primary research question regarding the impact of the physical activity program on HRQoL, a bivariate analysis will be conducted. The analysis will compare exposure to the physical activity program (intervention vs. control group) and the PedsQL survey scores at different time points (week 0, week 11, and week 24). For numerical variables with a normal distribution, the Student's t-test for independent samples will be used. For non-normally distributed variables, the Mann–Whitney *U* test will be applied. Additionally, to evaluate within-group changes over time (e.g., baseline vs. end of study), paired tests will be conducted using either the paired Student's t-test for normally distributed variables or the Wilcoxon signed-rank test for non-normally distributed variables.

An intention-to-treat analysis will be employed to ensure the robustness of the results and account for potential non-compliance or missing data. This approach will analyze participants based on their initial group assignment (intervention or control), regardless of adherence to the program or completion of all study components (22).

Statistical significance will be set at *p*-value < 0.05. All analyses will be performed using R Studio (R Project version 4.3.2).

### Patient recruitment

Patients will be recruited from Hospital SOLCA Quito and Hospital Pediátrico Baca Ortiz. These two hospitals account for approximately 65% of level 3 care and treatment services in the city of Quito, with most of their patients coming from the public health sector. The remaining services are divided among other institutions: approximately 15% are provided by private healthcare facilities, while the final 20% are distributed among several public hospitals that individually account for less than 7% of level 3 care (18).

**Figure 1:**
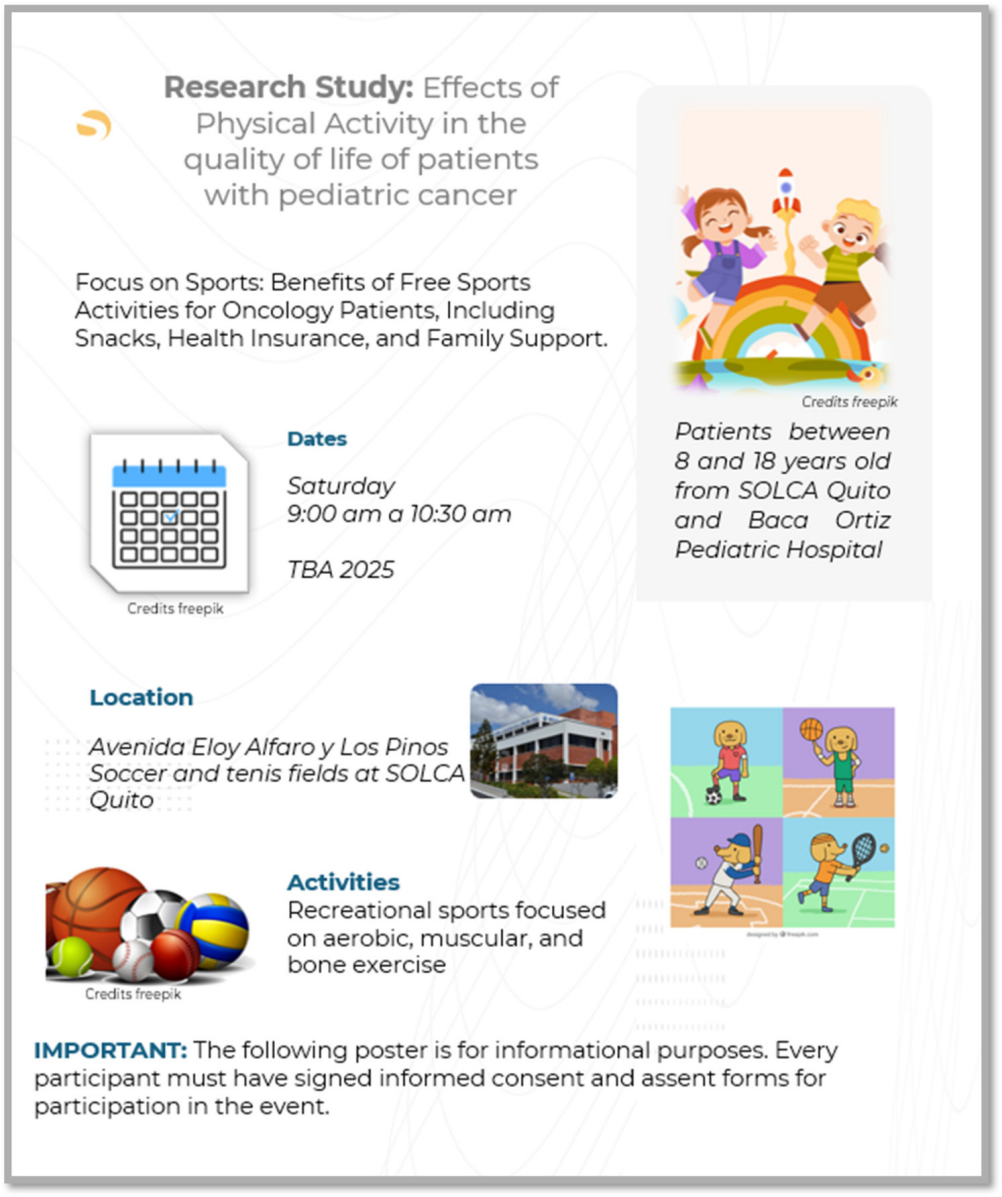
Promotional flier for recruitment of pediatric cancer patients for the physical activity program.

Recruitment will involve the distribution of a flyer (Figure 1) summarizing the project and inviting potential participants and their legal representatives to learn more. The flyer will also be shared digitally via mobile devices, allowing parents or guardians to review the project details at their convenience.

If parents or guardians express interest in learning more about the study, the medical oncologist will initiate the enrollment process by providing a detailed explanation of the project. Once the parent or guardian agrees to their child's participation, they will be required to read and sign the informed consent form and assent form. By signing these documents, parents or guardians authorize their child's medical oncologist to extract contact information and relevant diagnosis details from the medical records for inclusion in the study.

### Eligibility and randomization

This study will include participants aged 8–18 years. For study purposes, age groups will be categorized as follows:

• Children: Aged 8–12 years.
• Adolescents: Aged 13–18 years (14).

Participants will be randomized into one of two groups:

1. Intervention Group: Participates in a structured physical activity program.
2. Control Group: Does not participate in the physical activity program.

Randomization will be conducted using the REDCap randomization tool with allocation concealment ensured by restricting access to the randomization sequence (15). Medical oncologists involved in recruitment will not have access to group assignments at the time of enrollment. Additionally, the medical oncologist will be blinded for the group assignments, the social worker would know the group assignments for organization in participants for the physical activity program and surveys administered.

### Response to adverse events

Any foreseeable risk or adverse event to the participants will be classified under Gaub et Al. risks and adverse effects (16):

1. Grade 2–5: Any unfavorable and unintended sign, symptom, or disease temporarily associated with the use of a medical treatment or procedure that may or may not be related to the physical activity intervention.
  • Grade 2: minimal, local, or non-invasive intervention.
  • Grade 3: The condition is medically significant but not immediately life-threatening; hospitalization or prolongation of hospitalization is indicated; incapacitation and limited self-care are indicated.
  • Grade 4: Life-threatening consequences requiring urgent intervention.
  • Grade 5: An adverse event that culminates in death.

Grade 1: Any unintended or unfavorable sign or symptom not followed by a medical diagnosis or procedure that may or may not be related to the physical activity intervention. A single clinical or diagnostic observation occurs, and no intervention is indicated. For instance, symptoms such as shortness of breath, abdominal pain, disorientation, vomiting, bruising, headache, seizures, muscle pain, back pain, nausea, itching, and somatic reaction may be present.

### Adverse event reporting and program oversight

Grade 1 adverse events will be reported to the corresponding medical oncologist for participant evaluation. In the event of any adverse event classified as Grade 2–5, it will be reported to the designated medical oncologists, the Cancer Committee, and the Institutional Review Board (IRB) of the Ethics Committee at Universidad San Francisco de Quito (Quito, Ecuador) for further evaluation and to determine the continuity of the program.

The social worker, together with the principal investigator, will be responsible for monitoring and reporting any potential adverse events to the medical oncologists and the IRB/Ethics Committee, should they arise during the investigation. The oversight of the data and communication among the team members will be the responsibility of the data manager to ensure proper data collection, ethical data sharing and quality control procedures for data entry.

The physical activity program will be conducted at Hospital SOLCA Quito, with support from the physical activity team, as well as a brigade of nurses and emergency room physicians who will be available to provide immediate assistance in the event of any adverse incidents during the program.

### Sample size calculation

The sample size calculation for this study was conducted considering a confidence level of 95%, a power of 80%, equal variances between groups, and a 1:1 ratio between sample sizes. The difference in means to be detected was set at 4.36, based on literature identifying this value as the minimum clinically significant difference (17). The common standard deviation was calculated at 11.26, using the formula for the standard error of measurement (SEM = 15 × √1−0.85), where the Cronbach's alpha reliability coefficient is 0.85. The formula for hypothesis testing by comparing means of independent groups was applied using the Epidat 4.2 program, resulting in a required sample size of 106 participants per group. To account for an estimated 20% loss to follow-up, the sample size was increased to 133 participants per group. However, due to budgetary limitations, the final study sample will consist of a total of 90 participants (approximately 45 per group). While this is lower than the 160 participants initially estimated through power calculations, this adaptation reflects real-world constraints commonly encountered in resource-limited settings. The study is therefore best positioned as a pilot randomized controlled trial, with the aim of generating preliminary data to inform future larger-scale interventions.

### Inclusion criteria

1. Age range: Participants must be between 8 and 18 years old at the start of the study.
2. Residency: Patients residing within the city limits of Quito, Ecuador.
3. Treatment status:
  • Patients are being evaluated or treated at Hospitals SOLCA and Baca Ortiz.
  • Patients are either currently undergoing active treatment for pediatric malignancies, or
  • Patients are in the post-treatment phase after a pediatric cancer diagnosis.
  • The volunteer or a family member that lives with the possible participant has a cellphone so the survey can be administered and answered.

### Exclusion criteria

1. The presence of a malignant solid tumor which has not yet been surgically addressed.
2. A psychiatric diagnosis of cognitive impairment.
3. A diagnosis of osteosarcoma.
4. A history of cardiac pathology.
5. The patient is in the pediatric inpatient ward or was one month prior to the enrollment phase.
6. The patient is stationed at the pediatric intensive care unit.
7. The patient received high-dose chemotherapy with methotrexate greater than 1,000 mg/m^2^/dose or cytarabine greater than 1 g/m^2^/dose in the last month.

### Study duration

The study is expected to last approximately 18–22 weeks in total. Recruitment of participants will be conducted over an estimated 8–12 weeks. Following the identification of both the intervention and control groups, the 10-week structured physical activity program will begin for the intervention group, while the control group will proceed without any organized physical activity intervention (Supplementary Table S2).

## Discussion

Although the study sample size is constrained by budgetary limitations and is smaller than initially calculated, this research represents a valuable first step toward evidence generation in a neglected population and geographic context. The findings will contribute preliminary insights on the feasibility, safety, and potential effectiveness of a structured physical activity program in pediatric cancer care in Ecuador. These results may serve as a foundation for designing larger multicenter trials and for informing public health policy in similar low-resource environments. A key strength of this study is its use of repeated measures, which allows for the tracking of HRQoL changes over time at multiple points (week 0, week 11, and week 24). This approach provides a robust understanding of the temporal impact of the intervention, capturing both short- and long-term benefits. Additionally, this study is the first of its kind in Ecuador, addressing a critical gap in the literature on pediatric cancer care within the region. By focusing on a structured and accessible program, this study seeks to establish a scalable model that can be adapted to meet the needs of a broader pediatric population. The ultimate goal is to inform and enhance the design of care programs that are inclusive, sustainable, and capable of significantly improving the reach and quality of care for pediatric patients, particularly in resource-constrained settings.

This research serves as a foundational step for future studies and public health initiatives, encouraging further exploration of pediatric HRQoL not only in Quito but across Ecuador. By providing evidence-based insights into the effectiveness of physical activity programs, this study offers a critical framework for developing hospital-based interventions tailored to the unique needs of pediatric cancer patients. These findings have the potential to guide the expansion of such programs at a national level, addressing the disparities in pediatric cancer care in Ecuador and beyond.

The study also emphasizes the importance of interdisciplinary collaboration. By integrating expertise from diverse fields—such as public health, oncology, social work, physiotherapy, and physical education, the research underscores the necessity of a holistic approach to pediatric care. This collaborative model ensures that care programs address not only the medical but also the social, emotional, and physical dimensions of well-being. Such a comprehensive approach is essential for fostering resilience and a higher HRQoL for pediatric patients and their families.

## Limitations

This study acknowledges several limitations. First, budgetary constraints prevented the recruitment of the originally calculated sample size, potentially reducing the statistical power to detect small effect sizes. Second, the study was limited to participants residing in Quito, which excluded a substantial number of eligible patients from other provinces. Third, requiring participants or their families to have access to a cell phone may introduce selection bias and limit the generalizability of findings to lower socioeconomic groups. Fourth, given the nature of the intervention and the primary outcome measure (self-reported HRQoL), blinding of participants and outcome assessors was not feasible, which may increase the risk of response and measurement bias. However, data analysis will be conducted by a researcher not involved in intervention delivery to minimize this risk. Fifth, although intervention adherence will be monitored through attendance logs and fidelity ensured through a structured protocol, individual differences in participation may still influence results. Lastly, since the study was conducted in only two hospitals in Quito, the external validity of the findings may be restricted. Future studies should consider multi-site designs and additional monitoring strategies to improve generalizability and internal validity.

## Conclusion

This study represents a significant step in addressing the HRQoL challenges faced by pediatric cancer patients. By evaluating a structured physical activity program, it provides valuable evidence to support the integration of such interventions into routine pediatric oncology care. The findings will contribute to the growing literature on pediatric HRQoL, offering practical recommendations for enhancing care programs. Ultimately, this research aims to inspire future studies and collaborations that prioritize the well-being of children with cancer, fostering better outcomes and a brighter future for this vulnerable population.
