# Supplementary material for: Impact of a physical activity program on the health-related quality of life in pediatric cancer patients: a study protocol
Source: Front Sports Act Living. 2025 Jun 25;7:1559431. doi: 10.3389/fspor.2025.1559431 (PMC12239738; doi:10.3389/fspor.2025.1559431)
Supplement: Supplementary file 1 [file Table1.pdf]

Table S1. Roles and responsibilities.

**Role**	**Responsibility**
Medical oncologist	Evaluation of medical criteria and enrollment of participants
Social worker	Follow up and assistance for the registry of quality of life surveys.
Physiotherapists	Organization and implementation of physical activity program
Principal Investigator	Elaboration of the protocol, administration of budget, and elaboration of final work.
Physical education professional	Organization and implementation of physical activity program
Data manager	Proper data collection, ethical data sharing and quality control procedures for data entry.
Nurse and ER team	Provide support if any adverse event should arise during the physical activity program

Table S2. Study timeline.

**Activity**	**Duration**	**Description**
Recruitment	8-12 weeks before week 1	Recruitment of volunteers in Hospital SOLCA and Baca Ortiz. Registration of initial data (patient demographics), and randomization to occur.
Survey administration	Week 1	PedsQL surveys are sent via link texted to the participant's cell phone according to their age (8 to 12) and (13 to 18) by the social worker (SW). If the participant does not have a phone, it will be sent to the parent/guardian.
Physical activity Program	Weeks 2-11	Physical activity program for participants in physical activities
Survey administration	Week 11<br>After the 10-week program	PedsQL survey is texted to participant's (or parent/guardian) cell phone according to their age (8 to 12) and (13 to 18) by the SW.
Survey administration	Week 24<br>Three months after the end of the program (follow-up)	PedsQL survey is sent via link texted to the participant's (or parent/guardian) cell phone according to their age (8 to 12) and (13 to 18) by the SW.
